# Chitosan Modification and Pharmaceutical/Biomedical Applications

**DOI:** 10.3390/md8071962

**Published:** 2010-06-25

**Authors:** Jiali Zhang, Wenshui Xia, Ping Liu, Qinyuan Cheng, Talba Tahirou, Wenxiu Gu, Bo Li

**Affiliations:** 1 State Key Laboratory of Food Science and Technology, Jiangnan University, Wuxi, 214122, Jiangsu, China; 2 School of Medicine and Pharmaceutics, Jiangnan University, Wuxi 214122, Jiangsu, China; 3 School of Food Science and Technology, Jiangnan University, Wuxi 214122, Jiangsu, China; 4 Jiangsu Animal Husbandry and Veterinary College, Taizhou 225300, Jiangsu, China; 5 School of Chemical Engineering, Jiangnan University, Wuxi 214122, China

**Keywords:** chitosan derivatives, hypocholesterolemic, immunoenhancing, homeostasis, D-Glucosaminic Acid

## Abstract

Chitosan has received much attention as a functional biopolymer for diverse applications, especially in pharmaceutics and medicine. Our recent efforts focused on the chemical and biological modification of chitosan in order to increase its solubility in aqueous solutions and absorbability in the *in vivo* system, thus for a better use of chitosan. This review summarizes chitosan modification and its pharmaceutical/biomedical applications based on our achievements as well as the domestic and overseas developments: (1) enzymatic preparation of low molecular weight chitosans/chitooligosaccharides with their hypocholesterolemic and immuno-modulating effects; (2) the effects of chitin, chitosan and their derivatives on blood hemostasis; and (3) synthesis of a non-toxic ion ligand—D-Glucosaminic acid from Oxidation of D-Glucosamine for cancer and diabetes therapy.

## 1. Introduction

Chitosan, a natural cationic polysaccharide, has received considerable attentions as a functional, renewable, nontoxic and biodegradable biopolymer for diverse applications, especially in pharmaceutics [1], food [2] and cosmetics [3]. In the medical field, chitosan has been developed not only as artificial skin, absorbable surgical suture, and a wound healing accelerator, but also as new physiological materials due to their antitumor, immunoenhancing, antimicrobial and hypocholesterolemic properties [4]. These functions have been revealed to be dependent on both their chemical structure and the molecular size. As a result, the application of this native polysaccharide is limited by its high molecular weight and highly viscous nature resulting in its low solubility in acid-free aqueous media. In recent years, studies on the modification of chitosan have intensified since efficient utilization of marine biomass resources has become an environmental priority and for a better use of chitosan. This review focuses on the modification of chitosan by enzymatic hydrolysis and various chemical processes, in combination with pharmaceutical and biomedical applications especially in hypocholesterolemic, immunoenhancing, homeostasic and anticancer functions, based on our current research as well as the recent literature.

## 2. Structure and Characterization of Chitosan

Chitosan is a copolymer consisting of β-(1→4)-2-acetamido-D-glucose and β-(1→4)-2-amino-D-glucose units, with the latter usually exceeding 80%. The proportion of the two monosaccharide units in chitosan depends on the alkaline treatment Generally, the individual chains assume an essentially linear structure, which undergoes one full twist every 10.1–10.5 Å along the chain axis. Since each monosaccharide unit is chiral, the rotations of polymer chains show evident left or right. Accordingly, chitosan could be divided into three crystal types: α, β and γ type, which could be identified by X-ray model and NMR spectra. Among these, α-type is the most common type obtained from crust of shrimp and crab [5].

Chitosan has three types of reactive functional groups, an amino/acetamido group as well as both primary and secondary hydroxyl groups at the C-2, C-3 and C-6 positions, respectively. The amino contents are the main factors contributing to differences in their structures and physico-chemical properties, and its distribution is random, which make it easy to generate intra- and inter-molecular hydrogen bonds.

Many novel chitosan derivatives have been obtained by chemical modification using the reactive activities of hydroxy- and amino groups. In particular, the amino group has nucleophilic property, allowing easy formation of imine by reaction with aldehyde or corresponding amide derivatives in acylating reagents; in acidic solution, the amino groups showe alkaline properties and receive protons to generate salts, presenting cationic polymer properties. Besides, the amino functional group has also been correlated with chelation, flocculation and biological functions. The characterization of a chitosan sample requires the determination of its average Degree of acetylation (DA). The distribution of acetyl groups along the chain (random or blockwise) may influence the solubility of the polymer and also the inter-chain interactions due to H-bonds and the hydrophobic character of the acetyl group. This distribution has been evaluated by various techniques such as IR, elemental analysis, enzymatic reaction, UV, ^1^H liquid-state NMR and solid-state ^13^C NMR. Diad and triad frequencies were determined for homogeneous and heterogeneous chitosan with different values of DA [6].

Another important characteristic to consider for these polymers is the molecular weight and its distribution. The first difficulty encountered in this respect concerns the solubility of the samples and dissociation of aggregates often present in polysaccharide solutions. As to choice of a solvent for chitosan characterization, various systems have been proposed, including an acid at a given concentration for protonation together with a salt to screen the electrostatic interaction. The solvent is important also when molecular weight has to be calculated from intrinsic viscosity using the Mark-Houwink relation. High molecular weight chitosan is estimated to be 10^6^. High molecular weight chitosans (HMWC) form solutions with higher viscosities than chitosans of lower molecular weight. Polymeric chitosan is soluble in acidic conditions and is insoluble at pH values above 6.3 (the pKa of chitosan). However, chitosan oligomer has a low viscosity, and is freely soluble at neutral pH. Production of low molecular weight chitosan (LMWC) and chitooligosaccharides (COS) from the hydrolysis of chitosan can be achieved either by chemical or enzymatic methods. The chemical method needs high energy and is hard to control; hence, the enzymatic hydrolysis of chitosan offers many advantages. During preparation of different molecular weight chitosans, viscosity is used as a parameter to determine the molecular weight.

Unlike most polysaccharides, chitosan, LMWC and COS have positive charges, which allows them to bind strongly to negatively charged surfaces; this property is responsible for many of the observed biological activities [7]. However, it is important to mention that chitosans with different structures show different biological activities, and not all biological activities are found in one kind of chitosan. Each special type of bioactive chitosan has been developed by chemical modification and enzymatic hydrolysis for its potential pharmaceutical and medical application.

## 3. Enzymatic Preparation of LMWC and COS and Their Hypocholesterolemic and Immunoenhancing Effects

In general, for preparation of COS and LMWC, chitosan can be hydrolyzed enzymatically by specific chitosanases and non-specific enzymes. The specific enzyme for chitosan hydrolysis has been found in a wide range of organisms, including bacteria [8,9], fungi [10,11] and plants [12]. Most of these chitosanases are characterized as endo-type and they split β-1,4 glycosidic linkages in chitosan in a random way to form chitosan oligomers. However, the utility of these specific chitosanases in hydrolysis is limited because of its cost and unavailability in large quantities. Nowadays, on the one hand, kinds of physical/chemical induction and molecular evolution of special chitosanases have been attempted to obtain higher activity and stability [13]. On the other hand, many researchers have been devoting themselves to exploring novel sources of chitosanases [14–20]. Whereby, a number of non-specific commercial enzymes have been found for their ability to degrade chitosan with efficiency comparable to that achieved by chitosanases. Based on long-term work, we have made some achievements in both molecular evolution of special chitosanases from fungi and the chitosan-hydrolysis of these nonspecific enzymes as well as preparation of many kinds of LMWCs and COSs.

### 3.1. Non-Specific Enzymatic Preparations of LMWCs and Their Hypocholesterolemic Effects

#### 3.1.1. Non-specific enzymatic preparations of LMWCs

The reactions of non-specific chitosanolysis are of great interest as these commercial enzymes have been used in the food industry for decades [21–23]. They were found to be safe and relatively low-cost. Another advantage is the ease of production of LMWCs in higher yields due to their low specificity or nonspecificity. However, the nonspecific hydrolysis mechanisms are still disputed. Here we review the characterization and mechanism of chitosan hydrolysis based on the action of three typical non-specific enzymes: cellulase, lipase and papain.

##### 3.1.1.1. Cellulase-treated chitosan

Cellulase was one of the two nonspecific enzymes firstly reported with chitosanolysis properties and it also exerted comparable chitosanolytic activity with the specific chitosanases. There are various reports on chitosan hydrolysis by cellulases [24–27]. We also studied the characteristics and mechanism of cellulase treating chitosan. We were led to conclude that not a single chitosanolytic component existed in fungal commercial cellulases prepared from *Trichodrma viride*, exerting both exo- and endo-split pattern. The commercial cellulase could catalyze the hydrolysis of chitosan substrates with a wide range of acetyl groups and showed similar effects on chitosans with DA less than 30%. The optimal temperature for cellulase to function on chitosan was between 50 ºC and 60 ºC and the optimal pH was in the range of 5.0 to 7.0. The products of standard COS and chitosan digestion were analyzed by thin layer chromatography (TLC), Liquid chromatography mass spectrometry (HPLC/MS) and time course analysis. Results demonstrated that it could cleave GlcN-GlcNAc, GlcNAc-GlcN as well as GlcN-GlcN bonds from the non-reducing end of the chitosan chain [28,29]. Chitosan was extensively depolymerized into a range of molecular weights (Mw) from 286 kDa to 1588 kDa and deacetylated from 82% to 94%. Eight partial depolymerized chitosans were prepared [30]. About the mechanism, one bifunctional enzyme with chitosanolytic and cellulolytic activity (CCBE) from cellulase *(T. viride*) has been purified and further identified as a cellobiohydrolase I with exo-β-D-glucosaminidase activity, belonging to glycosyl hydrolysase seven families [31,32].

##### 3.1.1.2. Lipase-treated chitosan

Chitosan hydrolysis by lipase was firstly studied by Muzzarelli *et al.* [23]. In 2001, the degradation of chitosan was further studied with the aid of lipase from *Rhizopus japonicus* for the production of soluble chitosan, showing that lipase could degrade chitosan to water-soluble LMWC with Mw between 30–50 kDa at the optimal temperature of 40 ºC [33]. In a recent study, we investigated the effects of a commercial lipase from *A. oryzae* on chitosan hydrolysis systematically with different parameters such as pH, temperature, DA, Mw, viscosity reduction, and qualitatively analyzed COS products using kinetic analysis, TLC and HPLC methods. When four chitosans with various DA were used as substrates, the lipase exhibited higher optimal pH toward chitosan with lower DA. The optimal temperature of the lipase was 55 ºC for all chitosans. The enzyme exhibited higher activity to chitosans deacetylated at the level of 82.8% and 73.2%. Kinetics experiments showed that these two kinds of chitosan also had stronger affinity for the lipase. The chitosan hydrolysis carried out at 37 ºC produced larger quantity of COS than that at 55 ºC when the reaction time exceeded 6 h, and COS yield of 24 h hydrolysis at 37 ºC was 93.8%. Product analysis results demonstrated that the enzyme produced glucosamine and COSs with polymerization degree (DP) of 2–6 and above, and acted on chitosan in both an exo- and endo-hydrolytic manner. Moreover, one main chitosanolytic component with chitinase activity (CNBE) was purified from this commercial lipase (*A. oryzae*), and was identified as the exo-β-D-glucosaminidase with *N*-acetyl-chitobiosidase activity, belonging to glycosyl hydrolysase 18 family [34,35].

##### 3.1.1.3. Papain-treated chitosan

It has been reported that papain degrades chitosan under optimal the pH condition of 3.0–4.0 and temperature of 40–50 °C, although different papains were used in different researches [21,36,37]. Chitosan with Mw of 4–5 × 10^5^ was degraded by papain to produce the LMWC and COS in a range of 10^3^–10^5^. Chitosan depolymerization was reported by papain in the form of its lactate salt at acidic pH values [36]. Chitosans with average molecular weights in the range of 4–7 × 10^5^ could be easily depolymerized to highly polydisperse chitosans. Modified chitosans were also depolymerized, though with lower initial velocities. The molecular parameters of chitosan depolymerized with the aid of papain were also studied, revealing that preference was for chitosan with highest DP. The papain mainly cleaved the GlcN-GlcNAc bond in chitosan [37]. However, our previous research found that papain could split the GlcNAc-GlcN as well as GlcN-GlcN bond and produced the mixture of LMWC and COS with DP of 2–6 after 24 h hydrolysis. The optimal degradation conditions were at pH 4.0 and 40 ºC, taking 14.3% DA chitosan (0.5% concentration) as the substrate. Papain initially degraded quite quickly in the reaction, the rate of decline in viscosity was evaluated at 70.77% in 30 min then stabilized after 1 h. LMWCs with 4.1–56 kDa could be obtained [38]. Moreover, we discovered that papain has at least three hetero chitosanases responsible for its chitosanolytic activity.

#### 3.1.2. The hypocholesterolemic effects of different prepared LMWCs

Growing evidence indicates that chitosan can lower plasma and liver triacylglycerol (TG) as well as total cholesterol (TC) levels [39–43], exhibiting hypocholesterolemic and hypolipidemic effects. It has been reported that chitosan can reduce the risk of cardiovascular diseases [43] and has potent fat-binding capacity *in vitro* [44]. In addition, chitosan was also shown to increase fecal-neutral-steroid and bile-acid excretion in rats [39,41,43] and lower the postprandial plasma TG level in broiler chickens [45]. Among these, the hypocholesterolemic effect of chitosan was reported in humans for the first time by Maezaki *et al.* [40], they found that chitosan effectively decreased plasma lipid levels without side effects. However, controversy still exists surrounding the mechanism of the hypocholesterolemic and hypolipidemic effects of different chitosans. Taking into consideration that the DA and Mw of chitosan are two important characteristics that greatly affect its chemical and physiological properties, in combination with the state of chitosan, our recent work studied the effects of DA, Mw and particle size of different solid LMWCs prepared by commercial cellulase on hypocholesterolemia *in vitro* and *in vivo*.

The results of fat-, cholesterol- and bile-salts-binding capacities of different LMWC samples *in vitro* [30,44,46] indicated that the fat-binding capacity of LMWCs was significantly higher than that of cellulose, and it increased with increasing DA and Mw, while the cholesterol-binding capacity did not show significant variation with changes of DA and Mw, but was affected by the particle size. However, the bile-salt-binding capacity was greatly affected by Mw: the sample with the highest Mw showed the best binding capacity for bile salts, while the DA and particle size seemed to have no evident effect on the bile-salt-binding capacity. These results verified that the physicochemical properties of LMWCs affect its binding capacities and hypocholesterolemic and hypolipidemic activities *in vitro*.

The hypocholesterolemic effects of LMWCs with different physiochemical properties were further investigated *in vivo*. Rats fed diets containing the lowest-DA chitosan showed significantly lowered plasma triglyceride, total cholesterol and low-density-lipoprotein cholesterol (LDL-C) levels as well as elevated high-density-lipoprotein cholesterol (HDL-C) levels, although not all differences were significant. Moreover, the food-efficiency ratio also decreased with decreasing DA [46]. This is in conformity with the results observed by Deuchi *et al.* [47]. LMWCs with higher Mw limited the body-weight gain of adult rats significantly, reduced the food-efficiency ratio and lowered plasma lipids [46,48]. These results confirmed the effect of viscosity on hypocholesterolemic activity but also indicated that the viscosity was not the major factor influencing the hypocholesterolemic effects of chitosan in the upper gastrointestinal tract. Above a certain viscosity, the effect was small with increasing Mw. The particle size of LMWCs also evidently affected its hypocholesterolemic effect. LMWCs with a fine particle size effectively lowered plasma and liver lipid levels in rats [39]. In addition, the powdered form of LMWCs exhibited a greater rate of adsorption of oil than the flake type [49]. We also found that the particle size of LMWCs was the main property affecting its hypocholesterolemic effect. This is consistent with the report that powdered chitosans exhibited better cholesterol- binding capacity than cellulose, while chitosan in the flake form bound less cholesterol than cellulose [48].

Therefore, we can conclude that the physicochemical properties of LMWCs affect their hypocholesterolemic activities. The effects are more pronounced when the particles are finer in combination with lower DA and higher Mw.

#### 3.1.3. The hypocholesterolemic mechanism of LMWCs via adsorption, electrostatic force and entrapment

The hypocholesterolemic activity of LMWCs was higher when its DA was lower (90% deacetylated) at equal Mw and particle size; this might be due to the electrostatic attraction between LMWCs and anionic substances such as fatty acids and bile acids [50]. In addition, when fat and LMWCs are eaten together, the viscous LMWCs will entrap the fat droplets in the stomach. When the complex arrives at the small intestine, LMWCs precipitate together with the entrapped fat at neutral pH to prevent the digestion of fat; this has been proven *in vitro* [44]. When the DA and the particle size are comparable, the fat-binding capacity of LMWCs is enhanced with increasing Mw, suggesting that during the fat-binding process, the fat molecules are embedded in the long chain of chitosan; hence, a larger molecular weight means a longer chain and thus more fats will be embedded. Therefore, the electrostatic interactions with and entrapment of fat by the viscous polysaccharide chitosan, which would reduce the absorption of fat in the diet, was regarded as one factor in the fat-binding mechanism [46]. This factor also resulted in a better fat-binding capacity of chitosan than for cellulose. Moreover, at the same Mw, powdered chitosan has a smaller particle size, a higher total surface area and a more open pore structure than flake chitosan, facilitating adsorption. This suggests that the interaction between chitosan and bile salts as well as cholesterol is adsorption, which also contributes to its hypocholesterolemic effect. However, this adsorption function is likely weakened *in vivo* as chitosan can be dissolved in the acidic conditions of the stomach.

From the above, it is a conclusion that the combined effects of electrostatic attraction, embedding, adsorption and entrapment were the probable mechanisms of the hypocholesterolemic effects of LMWCs, which was further confirmed by the results of distribution and metabolism of LMWCs in rats through measuring the content of fluorescein isothiocyanate labeled chitosan (FITC-CIS) *in vivo*. Therefore, hypocholesterolemic mechanisms of LMWCs were identified as the combination of adsorption, electrostatic force and entrapment.

### 3.2. Enzymatic Preparations of COSs by Specific Chitosanases and Their Immuno-Modulating Effects

#### 3.2.1. Enzymatic Preparations of COSs by fungi specific chitosanases

The higher yield and activity of special chitosanases is a long-term pursued project. Until now, the highest activity chitosanases and the commercial chitosanase preparation are all produced from bacteria such as *Streptomycete*, most from which belong to the glycosyl hydrolysase 46 family. However, yields were low, resulting in high costs and limitations in application. Fungi are potentially an excellent source of chitosanases since they secrete chitosanase extracellularly, avoiding cytotoxicity and product inhibition.

*Aspergillus* has been reported broadly and is regarded as a powerful candidate for special chitosanase production [11,51–54]. In our previous study, *Aspergillus* CJ22-326—a fungi strain capable of utilizing chitosan as a carbon source—was isolated from soil samples. Two types of chitosanase (ChiA and ChiB) produced from the culture supernatant of *Aspergillus* CJ22-326 were purified to an apparent homogeneity, identified by SDS-PAGE through ammonium sulfate precipitation, CM-Sepharose FF chromatography, and Sephacryl S-200 gel filtration. Molecular weights of the enzymes were 109 kDa (ChiA) and 29 kDa (ChiB). Optimum pH values and temperature of ChiA were 4.0 and 50 °C, respectively, those of ChiB were 6.0 and 65 °C. Viscosimetric assay and analysis of reaction products of these enzymes, using chitosan as a substrate, by TLC indicated endo- and exo-type cleavage of chitosan by ChiB and ChiA, respectively. ChiA released a single glucosamine residue from chitosan and glucosamine oligomers. ChiB catalyzed the hydrolysis of glucosamine (GlcN) oligomers larger than a pentamer, and chitosan with a low DA (0–30%), and formed chitotriose with chitohexaose as the major products. Both of the activities of ChiA and ChiB increased proportionally with the DA decreasing of chitosan. In addition, many chemical and physical methods have been used to improve the CHiB activity [10].

Furthermore, in order to improve the chitosanolytic activity, recently, both ChiA and ChiB genes were cloned, site-directly mutated and expressed in *E. coli*. The recombinant ChiB exhibited higher chitosanolytic activity than previously reported fungal chitosanases [55,56]. The enzyme ChiB had a useful reactivity and a high specific activity for producing functional COS with high DP. Base on the amino acid similarity, ChiB exhibited high homology to other fungal chitosanases from *Fusarium solani* [57,58], which belonged to glycosyl hydrolysase 75 family.

In addition, we also utilized the chitosanase from *A. fumigatus* BSF114 to produce COSs with the DP of 3–7. Chitosan pentamer ((GLcN)_5_) and chitosan hexamer ((GLcN)_6_) were isolated and purified from COS by the ultra-filtration, nano-filtration, ethanol precipitation and the CM-Sephadex C-25 column. (GLcN)_5_ consisted of (GlcN)_4_ (59.84%) and (GLcN)_5_ (40.16%). (GLcN)_6_, however, mainly consisted of (GLcN)_6_ (93.11%) and (GLcN)_5_ (6.89%) [59]. These COSs were used for immunomodulating research.

#### 3.2.2. The immuno-modulating effect and mechanism of COSs

Previous studies showed that COS possesses not only some similar properties with chitin and chitosan, but also special physiological or functional properties that differ from chitin and chitosan [60]. Immune activities of COS have attracted interest among researchers [61]. A LMWC and a COS mixture isolated from this chitosan hydrolysate have different stimulatory effects on the cell proliferation and IgM secretion of the human hybridoma HB4C5 cells [62]. Oligochitosan consisting of chitohexaose has been reported to have a stimulatory effect on the release of interleukin 1β (IL-1β) and necrosis factor-alpha (TNF-α) in macrophages *in vitro* [63]. A quantity of investigations indicated that the immunoregulatory function of polysaccharide is due to the combination of polysaccharide and the lymphocyte surface receptor, namely complement III receptor (CR3). However, effect of the DP of COS on gene expression and secretion of cytokines was not understood. Herein, the immune mechanism of higher DP chitooligomers ((GLcN)_5_ and (GLcN)_6_) obtained by enzymatic hydrolysis of chitosan on gene expression was studied.

CR3 was initially described as an opsonic receptor. Subsequently, CR3-mediated lectin-sugar recognition mechanisms have been shown to play a major role in the phagocytosis of several pathogens [64]. Effects of (GLcN)_5_ and (GLcN)_6_ *in vivo* and *in vitro* on gene expression of cell surface CR3 receptor were investigated by relatively quantitative reverse transcription-polymerase chain reaction (RT-PCR) and Enzyme-linked immunosorbent assay (ELISA). The results showed that the expression of CR3 mRNA could be promoted by both (GLcN)_5_ and (GLcN)_6_. The promotion effect caused by (GLcN)_6_ was greater than that of (GLcN)_5_. The possible mechanism was that the polysaccharide bound the binding site of CR3 and changed the configuration of the receptor. The change of configuration can adjust the toxicity of target cells conditioned by iC3b. After binding to the CR3 receptor, (GLcN)_5_ and (GLcN)_6_ can activate phagocytes to improve the phagocytic ability and enhance the antibody transmission ability. Moreover, it can activate T cells and B cells and enhance their conjugated effect in the immune response. The molecular weight of (GLcN)_6_ is higher than that of (GLcN)_5_, and relatively, the amount of amino groups exposed of (GLcN)_6_ is also larger than that of (GLcN)_5_. Therefore, (GLcN)_6_ has more active binding sites to combine with the CR3 receptor surface of macrophage and lymphocyte. And also (GLcN)_6_ can change the configuration of CR3 receptor to show higher affinity for CR3 receptor [59].

Moreover, the effect of COSs on gene expression of important cytokines such as IL-1 and TNF-α in Mf as well as IL-2 and interferon-γ (IFN-γ) in lymphocytes were also studied using cell proliferation assay in our team. Our results indicated that (GLcN)_6_ and (GLcN)_5_ could promote the immune mediation of mice in relation with the gene transcription level of IL-1, TNF-α and IFN-γ as well as the protein translation level of these cytokines. The promotion effects caused by (GLcN)_6_ were greater than that of (GLcN)_5_ [65]. This result was in accordance with previous reports [66–68]. After mice were treated with chitin oligosaccharides or chitosan oligosaccharides, Mf were activated and adjusted mutually with other components of the immune system which hastened the production of reactive medium such as reactive oxygen and NO [67]. COSs could either directly kill pathogen micro-organisms or tumor cells by exerting an immune response, or enhance cytotoxic activity and then inhibit tumor cell production by activating T-cells, NK-cells and some other immune cells through some cytokines such as IL-1 and TNF-α [67,69]. TNF-α could exert synergetic effects and adjust the proliferation of Th1 cell systems together with IL-1 and IL-2 *in vitro* [69]. These results indicate that COS induced innate immune responses by up-regulating IL-1, TNF-α and IFN-γ and then played a role in immune functions of lymphocytes. This may be one of the molecular mechanisms for elucidating the immunity of COS.

## 4. Hemostasis Effects of Chitosan and Its Derivatives

### 4.1. Quality Requirements for Chitosan as a Hemostasis Material

Research showed that whole blood formed a coagulum rapidly upon exposure to chitosan [70]. The following studies have shown that chitosan acts as a hemostatic agent and may be used in various wound healing applications such as hemostatic bandages [71–73]. However, chitosan, only in its purest form, has an internal hemostatic dressing potentiality, as a drug delivery agent, tissue scaffolding and numerous other health related products [74,75]. As a natural product derived from the shells and components of organisms found in natural environments, chitosan carries a variety of contaminants, common to biologically derived materials such as protein and endotoxin, which varies from batch to batch, and thus must be carefully purified; subsequently, the resultant stimulatory immune responses in humans and animals is to be understood prior to medically related applications [76,77]. Recently, various chitosan-containing medical devices such as the hemostatic Celox for the treatment of bleeding, and bandages such as HemCon or QuikClot for the control of bleeding, are marketed in Europe and US [74]. These companies utilize processes that eliminate contaminants such as proteins, bacterial endotoxins and toxic metals, practicing Good Manufacturing Practice (GMP) guidelines. Besides, there are many reports on the preparation of high purity chitosan for medical grade through getting rid of the protein, lipid, metals and endotoxin [78–88] and chitins contaminated by metals are unsuitable for the preparation of medical grade chitosan.

### 4.2. The Hemostatic Effect of Chitosan and Its Derivatives

#### 4.2.1. The hemostatic effect of chitin and chitosan

Since Muzzarelli described the properties of chitin in human wounds in 1977 [89], a lot of research has been initiated all over the world into the hemostatic activities of chitin and chitosan [90–94]. The effects of chitin and chitosan suspensions (0.0001–1.0 mg/mL) on blood coagulation were evaluated by Okamoto *et al.*, the results of the blood coagulation time (BCT) showed that chitin and chitosan reduced BCT significantly in a dose-dependent manner, and they enhanced the release of the platelet derived growth factor-AB (PDGF-AB) and the transforming growth factor-β1 (TGF-β1) from the platelets, particularly with chitosan [90]. Even in a therapeutically anticoagulated (heparinized) rabbit model, chitosan treatment could effectively bring bleeding time within the normal range [91]. The same conclusion was reached when Wang *et al.* compared the hemostatic ability of chitosan and collagen sponge. For both chitosan and collagen sponges, the total amount of bleeding from the injured veins until hemostasis and the complete hemostasis success rates were similar. But the chitosan sponges strongly adhered to the surface of the rabbit muscles, whereas the collagen sponges were easily detached from the muscles [92]. A fly-larva shell-derived chitosan sponge (CS) was evaluated as an absorbable surgical hemostatic agent in a rat hepatic hemorrhage model [93], indicating that CS was a suitable implantable hemostatic material when compared to gelatin sponge or oxidized cellulose in both acute and chronic bleeding models. Recently developed internal chitosan bandages have been effective in achieving rapid hemostasis in large surgical and traumatic lacerations of the aorta, liver, lung, kidney and cardiac ventricles [94]. However, there is little research on the blood coagulation of chitosans with different physicochemical properties. Our recent study measured the *in vitro* coagulative activity of chitosan hydrochloride solution with different DA and Mw using tube and capillary tube methods. The results indicated that the DA and Mw of chitosan had great influence on the hemostasis of chitosan hydrochloride solution: this chitosan with higher DA and Mw exhibited better hemostatic activity, while the effect of DA and Mw was slight on the powder chitosan. From this, it can be deduced that the hemostatic mechanisms of chitosan in powder and hydrochloride solution were different. Meanwhile, organ damage *in vivo* experiments in rabbits showed that chitosan could promote rapid blood clotting at a wound injury in lung, spleen and kidney, and also reduce the amount of bleeding, exerting a good *in vivo* hemostatic effect.

#### 4.2.2. The hemostatic effect of chitosan derivatives

Based on the excellent hemostatic activity, lots of chitin and chitosan derivatives with different chemical or spatial structures have been explored. *N*,*O*-carboxymethylchitosan (NOCC) could decrease the whole blood clotting time (WHBCT) and lower the plasma recalcification time (PRT) value, similar to that of chitosan and chitin [95]. When used in a hypothermic coagulopathic grade V liver injury, Bochicchio *et al* found that a modified chitosan patch (MCP) could significantly reduce the post treatment blood loss and increase resuscitation mean arterial pressure (P < 0.0001 and P < 0.018, respectively) [96]. The photocrosslinkable chitosan (Az-CH-LA) is a chitosan hydrogel containing both lactose moieties and photoreactive azide groups. The sealing ability of this chitosan hydrogel expressed stronger similarity to that of fibrin [97]. The photocrosslinkable chitosan has been used for endoscopic cancer treatment in the alimentary tract [98]. It could completely stop the bleeding from a cut mouse tail within 30 s of UV-irradiation and could firmly adhere two pieces of sliced mouse skin to each other. Its hemostatic effect is independent of the blood coagulation [99]. Moreover, a series of new *in situ*-forming hydrogels, composed of oxidized dextran (Odex) and amine-containing polymers including polyallylamine (PAA), oligochitosan and glycol chitosan, have been developed [100]. Some scholars also explored chitosan derivatives with the amino group introduction by using Ar, O2, NH3 and NH3-He mixed gas plasmas [101]. These samples all showed shorter blood clotting time, while the best was with treatment by NH3 and He plasma with O2 pretreatment.

Among these, chitosan-Glycerol Phosphate hydrogels were the most attractive, and have been deeply developed [102,103]. Chitosan-glycerol phosphate (chitosan-GP) is a unique polymer solution that is mixed with whole blood and solidified over microfractured or drilled articular cartilage defects in order to elicit a more hyaline repair cartilage [104]. It was found by Buschmann [102] that chitosan-GP/blood clots showed increased adhesion to the walls of the defects as compared with the blood clots in the untreated microfracture defects. After histological processing, all blood clots in the control microfracture defects had been lost, whereas chitosan-GP/blood clots adhered to and were partly retained on the surfaces of the defect. At six months, defects that had been treated with chitosan-GP/blood were filled with significantly more hyaline repair tissue (p < 0.05) compared with control defects. Repair tissue from medial femoral condyle defects that had been treated with chitosan-GP/blood contained more cells and more collagen compared with control defects and showed complete restoration of glycosaminoglycan levels. These results indicated that solidification of a chitosan-GP/blood implant in microfracture defects improved cartilage repair compared with microfracture alone by increasing the amount of tissue and improving its biochemical composition and cellular organization. Later, they further studied the effects of the chitosan-GP/blood implant and of debridement on the formation of incipient cartilage repair tissue by histology and *in vitro* clot retraction tests [103]. They found that chitosan-GP solutions structurally stabilized the blood clots by inhibiting clot retraction. Chitosan-GP/blood implants applied in conjunction with drilling, compared to drilling alone, elicited a more hyaline and integrated repair tissue associated with a porous subchondral bone replete with blood vessels. Concomitant regeneration of a vascularized bone plate during cartilage repair could provide progenitors, anabolic factors and nutrients that aid in the formation of hyaline cartilage. In addition, a series of chitosan-polyphosphate dressings were fabricated and the optimal chitosan-polyphosphate formulation (coded as ChiPP) accelerated blood clotting (p = 0.011), increased platelet adhesion (p = 0.002), generated thrombin faster (p = 0.002), and absorbed more blood than chitosan (p < 0.001) [106].

Based on the hemostatic properties reported from studies of chitin, chitosan and their derivatives, commercial chitin- and chitosan-based hemostatic dressings have flooded the market (see Table 1) and are used in medicine.

### 4.3. The Hemostatic Mechanism of Chitosan and Its Derivatives

Due to the effects of chitosan and its derivatives on hemostasis, there are lots of reports on studies of the mechanism *in vitro* and *in vivo*. Although there is still no assured verdict of the mechanism by which they function to stop bleeding, several factors are thought to contribute to the hemostatic function.

#### 4.3.1. The hemostatic mechanism of chitosan

Studies indicate that chitosan’s hemostatic mechanism seems to be independent of the classical coagulation cascade [110]. It has been found that coagulation factors could not be activated by chitosan and its derivatives [111], and no activation of the intrinsic pathway was observed by Benesch *et al.* [112]. Chitosan’s putative capacity to induce clot formation in the absence of coagulation factors could prove to be useful for patients with coagulopathies or those who are therapeutically anticoagulated, since chitosan keeps ability to maintain hemostasis in the presence of heparinized blood. Most of all, numerous articles reported the interactions between chitin/chitosan and platelets [113–115]. These studies suggested that chitin and chitosan directly influenced platelets by themselves and this effect was enhanced in the presence of plasma. Some researchers regarded that chitosan likely function independently of platelets because it could induce clot formation in the absence of platelets [111,116], however, over time, more and more researchers admit that the hemostatic effects of chitosan are related to both platelets and erythrocyte aggregations. The blood clot formed in the chitosan-treated lingual incisions was evaluated by scanning electron microscopy, and it showed an interesting phenomenon: when the blood mixed with chitosan acetic-acid physiological saline solution, the erythrocytes aggregated and were deformed. The deactylation degree in the chitosan acetic acid physiological saline solution - especially a low deactylation degree - had a significant effect on the unusual aggregation and deformation of erythrocytes, compared with the effect of Mw within a range between 10^5^ and 10^6^. However, this phenomenon could not be observed in solid-state chitosan soliquoid [116]. Our recent research also demonstrated that the hemostasis of chitosan has no direct relation to the traditional intrinsic or extrinsic pathways, but it could promote the adhesion and aggregation of platelets. Platelets play a very important role in coagulation and could promote hemostasis and thrombosis. The membrane space configuration changes and the platelet-derived microparticles (PMPs) are released in the activated platelets, then the exposed platelet glycoprotein IIb-IIIa receptor complex interacts with fibrinogen, resulting in platelet accumulation, and ultimately initiating the chain reaction of coagulation. Binding or agglutination of red blood cells in the presence of chitosan is dependent on its physical characters, particularly on molecular size. The ionic attraction between negatively charged red blood cell membranes and positively charged groups in chitosan is a possible explanation for the anticoagulant activity of chitosan. Therefore, positively charged chitosan is more effective than chitin as a blood coagulant [113].

Except for platelets and erythrocytes, chitosan also accelerated thrombin generation in a statistically significant manner compared to the saline control, with a trend towards chitin being more thrombogenic [117]. Upon acetylation, the chitosan layer became a strong activator of the alternative pathway of the complement system [115].

#### 4.3.2. The hemostatic mechanism of chitosan derivatives

Except for the hemostatic mechanism of chitosan itself, the hemostatic responses to its derivatives highly depend on their chemical nature such as the added functional groups. Hence, different chitosan derivatives are involved in different hemostatic mechanisms. For example, carboxymethyl chitosan physiological saline solution had nothing to do with the aggregation and deformation of erythrocytes but caused local rouleau [115]. The morphology of red blood cells binding surface on chitin and chitosan was examined, with approximately 10-fold lower levels and with less distinct general morphologies than the highly structured β-pGlcNAc [118].

Chitosan-glycerol phosphate (chitosan-GP) is one of the most popular chitosan derivatives and underwent detailed research by the company Buschmann and Biosyntech through investigating the hemostatic mechanisms underlying chitosan-GP/blood implant solidification *in vitro* and *in vivo* [103–105,119]. They found that chitosan-GP/blood clots solidified in an atypical biphasic manner, with higher initial viscosity and minor platelet activation followed by the development of clot tensile strength concomitant with thrombin generation, burst platelet and FXIII activation. Whole blood and chitosan-GP/blood clots developed a similar final clot tensile strength, while polymer-blood clots showed a unique, sustained platelet factor release and greater resistance to lysis by tissue plasminogen activator. Thrombin, tissue factor (TF), and recombinant human activated factor VII (rhFVIIa) accelerated chitosan-GP/blood solidification *in vitro* (P < 0.05). Pre-application of thrombin or rhFVIIa + TF to the surface of drilled cartilage defects accelerated implant solidification *in vivo* (P < 0.05). In conclusion, chitosan-GP/blood implants solidify through coagulation mechanisms involving thrombin generation, platelet activation and fibrin polymerization, leading to a dual fibrin-polysaccharide clot scaffold that resists lysis and is physically more stable than normal blood clots. Clotting factors have the potential to enhance the practical use, the residency, and therapeutic activity of polymer-blood implants [119].

## 5. Synthesis of D-Glucosaminic Acid by Oxidation from D-Glucosamine: A Useful Metal Chelate for Anticancer and Anti-Diabetic Purposes

D-glucosamine is a structural unit of chitosan, and is produced commercially by the hydrolysis of chitosan in hydrochloric acid. D-glucosamine could be oxidized to D-glucosaminic acid. D-glucosaminic acid is one of the carbohydrate units used to manufacture various biotic substances. It has various physiological functions.

### 5.1. Biological Activities of D-Glusosaminic Acid

In recent years, research on D-glucosaminic acid has increased because of its industrial, agricultural, food and medical applications. D-glucosaminic acid has recently been identified as a promising sweetener and condiment [120]. It is also a member of the “chiral pool” and has been used as a starting material for the asymmetric synthesis of various amino acids and several glycosidase inhibitors [121,122]. In addition, D-glucosaminic acid has recently been investigated as an unusual component of *Rhizobium leguminosarum* lipopolysaccharide [123], and its use as a cation coordinating agent has been widely studied [124]. Among these, the most remarkable was investigation of D-glucosaminic acid as a biocompatible, non-toxic ligand chelated with many metals for potential medical applications.

D-glucosaminic acid iron (III) complexes are potential pharmaceuticals in human and veterinary iron therapy since their stabilities are high enough to prevent metal ion hydrolysis in biological systems. Water-dispersed magnetic nanoparticles were successfully developed through glucosaminic acid-surface modification of iron oxide nanoparticles and its anticancer effect was then studied by Yu *et al.* [125]. The resultant glucosaminic acid-modified magnetic nanoparticles (GA-MNPs) had not only good uniformity in spherical shape with diameter of about 10–13 nm, but also possessed excellent water-dispersal and stability. In cell culture experiments, the internalization of GA-MNPs into different kinds of cells was observed over a five-day period. The results indicated that the internalization of GA-MNPs into mouse macrophage cells and mouse embryonic fibroblast cells was not observed after 40 h of culturing. However, the GA-MNPs were internalized quickly into cancer cells after just 24 h of culturing. TEM images of the GA-MNPs uptake in ECA-109 cells were used to study the internalization mechanisms of GA-MNPs and their distribution in ECA-109 cells. Besides this, the platinum(IV)/D-glucosaminic acid complex has received considerable attention for cancer therapy because of lower toxicity and the possibility of oral administration [126].

Moreover, D-glucosaminic acid has also been selected to design a new kind of chromium drug candidate for anti-diabetic purposes [127]. Two chromium (III) 1:1 and 2:3 (Cr: glucosaminate) complexes of glucosaminic acid were synthesized by neutralization and exchange reaction. The effect of the complexes on decreasing blood sugar was investigated on type-2 diabetes model rats induced by tetraoxypyrimidine. The results indicated that the effect on decreasing blood sugar was comparable to that of picolinate chromium complex (Cr (pic)3) currently used worldwide.

### 5.2. Synthesis of D-Glucosaminic Acid by Oxidation from D-Glucosamine

**Figure 1:**
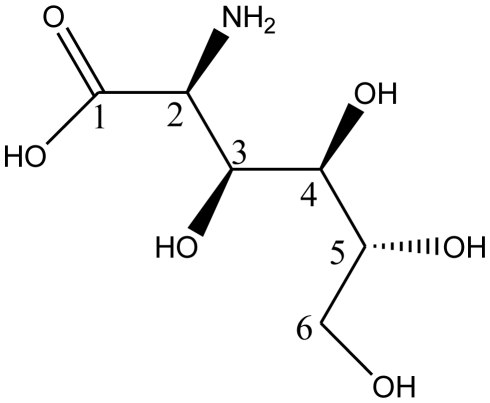
The molecular structure of D-glucosaminic acid.

The molecular structure of D-glucosaminic acid [128] is shown in Figure 1. Three synthesis strategies have been reported for D-glucosaminic acid from D-glucosamine: metallic catalytical oxidation, electrocatalytical oxidation and biosynthesis methods. A summary of the methods is given in the following context.

#### 5.2.1. Metallic catalytic oxidation method

##### 5.2.1.1. Oxidations with HgO and Hg(Ac)_2_

The classical synthesis of D-glucosaminic acid from D-glucosamine involves the use of yellow mercuric oxide as an oxidant [129], or mercuric acetate [130], followed by treatment with hydrogen sulfide, in a totally non-ecological process. One century ago, the yields of these procedures were not consistent (about 54%) and the resulting mercuric sulfide was difficult to separate from the products, which limited applications of D-glucosaminic acid in the food and pharmaceutical industries. However, recently we chose one novel separation method, the process was effective in a short time and the purity was higher than 95%. The yield of production through this process was estimated at a minimum of 50% of the amount of D-glucosamine chloride in use.

##### 5.2.1.2. Platinum and Palladium Catalyzed Oxidations

The oxidation of mannose over a platinum black catalyst was the first application in the field of carbohydrates [131]. Subsequently, the catalytic oxidation of carbohydrates over platinum on carbon was extensively studied [132–134]. Like platinum, the related noble metals palladium and rhodium are known to catalyze alcohol oxidations with O_2_. The oxidation of carbohydrates catalyzed by platinum and palladium has been reviewed a number of times [133]. D-glucosamine can also be oxidized by O_2_ with platinum and palladium catalysts, as can be seen in Table 2.

**Figure 2:**
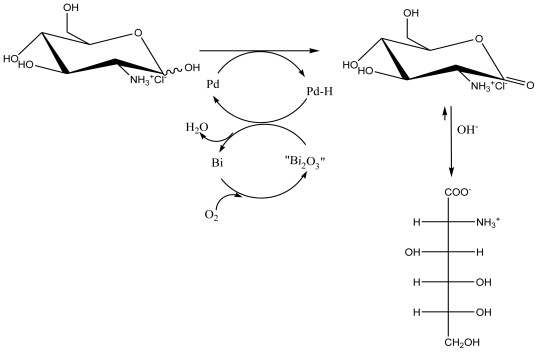
Tentative scheme for the mechanism of D-glucosamine hydrocloride oxidation on Pd-Bi/C catalyst [128].

Based on our recent research and the literature, we concluded that the noble metal catalyzed oxidation of D-glucosamine to D-glucosaminic acid is essentially an oxidative dehydrogenation reaction, like alcohols and aldehydes (see Figure 2). The substrate is dehydrogenated by the noble metal, followed by oxidation of the adsorbed hydrogen atoms. Poisoning of the catalyst by molecular oxygen occurs by formation of sub-surface platinum or palladium oxide resulting from oxygen adsorption of the surface in the absence of a reducing agent. This poisoning can be limited using a low oxygen pressure but cannot be precluded entirely. However, the catalyst can be regenerated using hydrogen as the reducing agent to regain the original activity. Otherwise, this poisoning can be precluded entirely by using both Pd and Pt catalysts doped with bismuth, where bismuth acts as an assistant catalyst, avoiding the overoxidation of the palladium and platinum surface. For this reason, the yield of D-glucosaminic acid with active charcoal supported Pd-Bi catalyst is much higher than with the other catalysts. Moreover, the preparation of D-glucosaminic acid by use of molecular oxygen as an oxidant and Pd-Bi/C as a catalyst presents some additional advantages, namely, (i) high selectivity and mild reaction conditions (oxidation with molecular oxygen at atmospheric pressure near room temperature); (ii) the possibility of conducting the oxidation in one step in a single reaction vessel; (iii) a higher site-time yield per catalyst mass; (iv) a much shorter reaction time (3 h). Moreover, the catalytic process is environmentally clean since it is conducted on recyclable catalysts and gives no noxious effluents or side products. Therefore, this method is more attractive for the oxidation of D-glucosamine [128]. The method further aims to ensure easy removal of the catalyst from the reaction mixture and to enhance its mechanical stability, as well as to enable operation in a fixed or suspended bed.

#### 5.2.2. Biosynthesis methods

Two main strategies including enzymatic and microbiological methods are used to synthesize D-glucosaminic acid from D-glucosamine. A combination system of glucose oxidase (EC 1.1.3.4) and Catalase (EC 1.11.1.6) has high catalytic activity (yield: 76%) in an aqueous solution of pH 7 [135]. It has been reported that some microbes, such as *Acinetobacter sp*., *Aerobacter sp*., *Acetobacter sp*. and *Escherichia sp*., produce D-glucosaminic acid from D-glucosamine [136–141]. As to enzymatic oxidation, D-glucosaminic acid was prepared by air oxidation of D-glucosamine catalyzed by glucose oxidase. However, glucose oxidase accepts D-glucosamine only as a poor substrate; the maximal catalytic efficiency was 2%. Thus, a better yield of D-glucosaminic acid was dependent on larger amounts of enzyme conjugated to longer reaction times (72 h).

Biosynthesis methods include many more procedures, such as culturing of strains, screening of strains, sterilization, *etc*. Currently, strict temperature controls, more by-products, longer reaction times, and lower purity of products limit the development of these methods. Therefore, the development of these methods requires many technological problems to be solved.

#### 5.2.3. Electrocatalytical oxidation

Electrocatalytical oxidation of D-glucosamine was first reported by Yao *et al.* [142]. The authors discussed the oxidation of glucose and derivatives at platinum black electrodes in pH 7.4 saline solution (isotonic with human plasma) with the objective of determining the feasibility of consuming such materials in an implantable energy conversion device. The anodic process would be oxidation of carbohydrates at an electrode with an associated diffusion membrane to prevent contact with blood or intercellular fluid, and the cathodic process would be reduction of oxygen. The catalytical effects of different electrode materials on the anodic oxidation process were further researched by Appleby and Drunen [143]. The results obtained indicate that platinum, rhodium and Pt-Ru alloy electrodes are effective for glucose and D-glucosamine oxidations. In 2007, Tominaga *et al.* [144] fulfilled the synthesis of D-glucosaminic acid using the novel approach of the electrocatalytic oxidation of D-glucosamine using gold electrodes and gold nanoparticle-modified carbon electrodes. The cyclic voltammetric results obtained indicated that D-glucosamine was catalytically oxidized in alkaline and neutral solutions. The controlled-potential electrolysis of D-glucosamine was possible in both alkaline and neutral solutions, and D-glucosaminic acid was identified as the 2-electron oxidation product. In particular, in an alkaline solution at a potential of-0.2 V, D-glucosaminic acid was formed as the 2-oxidation products with a current efficiency of 100%. When compared to the conventional synthesis method of D-glucosaminic acid, this synthesis approach is a useful alternative.

Although the electrocatalytical oxidation method overcomes the disadvantages of biosynthesis methods, such as more by-products and complicated processes, it is seldom adopted in industrial produce due to the higher energy consumption and the difficulty to control conditions.

In a word, although much research has been done on the synthesis of D-glucosaminic acid by metallic catalytical oxidation, electrocatalytical oxidation and biosynthesis methods, industrialized synthesis of D-glucosaminic acid has not yet been realized. The biosynthesis method produces higher yields, but some technological problems must be solved regarding the complicated procedures, such as separation of products, culture and screening of strains, *etc*. The noble-metal catalyzed oxidations with carbohydrates and derivatives have received increasing attention in recent years. In a number of cases, the selectivity of these catalytic processes can match those of enzymatic processes with the additional advantages of higher site-time yields and cleaner technologies. Thus, this method has received more and more attention. It is a promising synthesis method for D-glucosaminic acid in industry. However, there are still some parameters to be clarified in order to fulfill the necessary requirements for industrial development, such as an appropriate preparation system for catalysts and improvement of activity, stability and selectivity of catalysts.

## Figures and Tables

**Table 1:** Some commercial hemostatic dressings based on chitosan [104, 105, 107–109].

Commercial name	Company	Material and function
HemCon®	HemCon	Freeze-dried chitosan acetate salt, for emergency use to stop bleeding
Chitoflex®	HemCon	Based on chitosan, antibacterial, biocompatible wound dressing designed to be stuffed into a wound track to control moderate to severe bleeding
Chitoseal®	Abbott	Based on chitosan, backed with cellulose coating, for bleeding wounds
Clo-Sur®	Scion	Based on chitosan, a pressure pad applied topically to accelerate wound healing
TraumaStat®	Ore-Medix	Freeze-dried chitosan containing highly porous silica
Syvek-Patch®	Marine Polymer Technologies	Made of fully acetylated, high molecular-weight chitin in a crystalline, three-dimensional beta structure array, and isolated from the centric diatom Thalassiosira fluviatilis. It is claimed to be 7 times faster in achieving hemostasis than fibrin glue, because it agglutinates red blood cells, activates platelets whose pseudopodia make robust contact with chitin and promotes fibrin gel formation within the patch, thus acting in a redundant way even on heparinized patients
BST-CarGel®	Biosyntech company	chitosan-glycerophosphate hydrogels, a biodegradable gel for cartilage repair

**Table 2:** The oxidation of D-glucosamine to D-glucosaminic acid by (promoted) noble metal catalysts [128].

Catalyst	Yield
PtO2	37%
Pd	54–60%
Pd-Bi (yield: 70%)	70%
